# Coping in the Covid-19 pandemia: how different resources and strategies can be risk or protective factors to mental health in the Brazilian population

**DOI:** 10.1080/21642850.2021.1897595

**Published:** 2021-03-12

**Authors:** Fernanda de Oliveira Ferreira, Júlia Beatriz Lopes-Silva, Gustavo Marcelino Siquara, Edi Cristina Manfroi, Patrícia Martins de Freitas

**Affiliations:** a Basic Sciences Department, Federal University of Juiz de Fora, Governador Valadares, Brazil; b Psychology Department, Federal University of Minas Gerais, Belo Horizonte, Brazil; c Bahia School of Medicine and Public Health, Salvador, Brazil; d Multidisciplinary Health Institute, Federal University of Bahia, Vitória da Conquista, Brazil

**Keywords:** Stress, depression, anxiety, coping, social distance

## Abstract

**Objective:**

The present study's objective was to investigate the pandemic's impact on mental health and identify variables that can increase or decrease the chances of stress, anxiety, and depression, in both a sample with and one without self-reported mental health issues, in a Brazilian population.

**Design:**

a cross-sectional quantitative study. Data were collected online in May and June of 2020. Participated 1130 adults between 18–78 years old (mean = 37.46 years, SD = 12.18), from 20 Brazilian states, with an average of 58.61 days (SD = 23.2) of social distancing.

**Main Outcome Measures:**

Depression, anxiety and stress symptoms, characterization of social distancing, and Coping strategies.

**Results:**

A significantly higher prevalence of severe depression was found in those who practiced social distancing. Multinomial logistic regressions identified the explanatory model with risk and protection variables to mental health. For the group without a previous mental health diagnosis, using confrontation (*OR *= 1.39, *CI95%* 1.23–1.58) and escape strategies (*OR* = 1.48, *CI95%* 1.19–1.84) increases the odds of presenting severe depression, while positive reappraisal (*OR* = 0.85, *IC95%* 0.78–0.93) and problem-solving (*OR* = 0.75, *CI95%* 0.63-–0.88) were protective factors. In the group with mental disorders, using confrontation (*OR* = 1.33, *CI95%* 1.10–1.60) and escape strategies (*OR* = 1.49, *CI95%* 1.12–1.98) were also risk factors for severe depression and no coping protective factors were found.

**Conclusions:**

Problem-solving and positive reappraisal were protective strategies that potentially reduced the odds of presenting depression and anxiety, but only in people without a previous mental health diagnosis. Public policies must offer psychological support to the most vulnerable, as well as orientation based on scientific evidence, aiming at improving quality of life.

## Introduction

The new coronavirus, COVID-19, was considered by the World Health Organization as a Public Emergency in January of 2020, and it was recognized as a pandemic with a considerable impact on mental health and psychosocial well-being (Wang, Xia, Xiong, Li, Xiang, Yuan, et al., 2020). It is undeniable that the COVID-19 pandemic leads to a wide range of psychiatric symptoms in the global population (Xiong et al., 2020). The current context of unpredictability, uncertainty, the seriousness of the disease, misinformation, and social isolation can undoubtedly lead to stress and mental disorders (Le et al., 2020; Tran et al., 2020 Wang, Pan, Wan, Tan, Xu, Ho, et al., 2020a; Wang, Pan, Wan, Tan, Xu, McIntyre, et al., 2020b;). Rajkumar (2020) reviewed adverse mental health consequences and found that symptoms of anxiety and depression (16–28%) and self-reported stress (8%) are commonly associated with the COVID-19 pandemic.

Dealing with the distress of infectious diseases imposes a challenge to the maintenance of well-being. The investigation of coping strategies can be a better framework to understand the risk and protective factors of mental health issues. For instance, to decrease negative adjustment (e.g. depressive symptoms), it might be useful to reduce negative coping strategies, such as self-blame (Heffer & Willoughby, 2017).

Stress is defined as an overload due to situations that exceed the resources needed for coping. Stressor’s presence triggers the coping strategies that emerge to reduce or eliminate the adverse effects of stress (Lazarus & Folkman, 1984). Faced with inefficient strategies, the stress levels increase and have consequences for physical and mental health (Folkman & Lazarus, 1980). This relationship between the overload triggered by the stressor, the low effectiveness of coping strategies, and mental health damage has been recognized as a bidirectional mechanism that establishes a feedback cycle (Endler, & Parker, 1990). In addition to the bidirectionality between stress and mental disorders, it is necessary to consider that the relationship between stress and coping is influenced by different variables, thus being a mechanism driven by a multifactorial system with effects of socioeconomic aspects, personal and social resources.

Coping strategies are divided into two different focuses: emotion-focused coping and problem-focused coping, and each category has specificities and distinct effects (Folkman & Lazarus, 1985)). Emotion-focused coping is any strategy used to reduce stress by regulating the emotional state. Its primary function is to reduce tension, so it is also recognized as a palliative mechanism. On the other hand, problem-focused coping consists of actions that resolve or modify the stressful situation and eliminate the stressful event (Lazarus & Folkman, 1984; Folkman, Lazarus, Dunkel-Schetter, DeLongis, & Gruen, 1986).

The application of coping strategies is associated with reducing stress and improving the emotional state. As for functionality, researchers claim that reducing stress depends on who, when, and under what circumstances coping is being used (Lazarus & Folkman, 1987). The mechanism for assessing the situation and the possibilities for reducing stress is crucial to how effective a strategy can be. For example, acceptance and resignation can be very adaptive in situations without control. Likewise, positive thinking and positive reevaluation without a plan that involves significant efforts to change stressful situations can be a threat to mental health (Folkman et al., 1986; Lazarus & Folkman, 1987). Regarding COVID-19, it is possible to consider that the pandemic has a collective stressor effect because it represents a threat to health, socioeconomic situation, illness, and family and friends’ death.

Chew, Wei, Vasoo, Chua, and Sim (2020) summarized psychological responses and coping methods during previous outbreaks of infectious diseases, such as Ebola and H1N1. Chew et al. (2020) described that the most frequently found coping strategies were problem-focused coping (such as seeking alternatives, self-, and other-preservation), seeking social support, avoidance, and positive reappraisal of the situation. The coping strategy varied in different pandemics, being the problem-focused strategies more prone to predict better adjustment in controllable situations, whereas emotion-focused strategies (such as avoidance and denial) would be favored in uncontrollable situations.

Regarding the current COVID-19 pandemic, Gerhold (2020) conducted a representative survey with German citizens focusing on the perception of risk and ways of coping. Findings from this study show that participants were concerned about COVID-19 but not about getting infected. Regarding the assessment of problem-focused and emotion-focused coping strategies, while facing the COVID-19 pandemic, it was reported that problem-focused strategies, such as ‘listening to experts’ and ‘not trying to do anything rash,’ were the most common. Nevertheless, this study did not evaluate coping strategies specifically associated with mental health issues. It is relevant to investigate the psychological impact and ways to cope in different population groups to allow for a more comprehensive understanding of the COVID-19 impact on mental health.

A specific group that should be further investigated in more in-depth detail is the one that already has previous mental health conditions. The pandemic has led to a generalized state of anxiety, depression, and stress, but people with mental health conditions could be more prone to being influenced by these emotional responses due to a higher susceptibility to stress (Hao et al., 2020). This could cause relapses or worsen their preexisting conditions (Yao, Chen, & Xu, 2020). Besides that, this group is also more vulnerable to get infected by the virus or to have a worse prognosis due to high rates of smoking (Druss, 2020). Finally, the lockdown policies could potentially disrupt mental health services and treatments because mental health institutions can potentially become epicenters for infection due to challenges in implementing physical distancing (Moreno et al., 2020).

Dong and Bouey (2020) emphasize that, especially in countries with a high number of COVID-19 cases, there could be a mental health crisis, and this requires the need to incorporate mental health care in disaster management plans. Understanding the mechanisms underlying this high rate of impact on mental health during the COVID-19 pandemic is hugely relevant to developing novel interventions to protect mental well-being (Holmes et al., 2020).

The first COVID-19 case in Brazil was confirmed on February 26th. At the beginning of November 2020, Brazil’s total number of COVID cases was 5,567,126, with 160,548 deaths. Since then, measures to contain the spread of the virus have been adopted in the country. Brazil is a country with continental dimensions, and there was no unified policy to control the dissemination of COVID-19: states and cities vary widely regarding the adopted health-protective measures. An example is the different levels of partial lockdown that were proposed by the city councils, as well as the inspection concerning the use of masks. Social distancing measures have started in March. It is worth mentioning Brazil’s cultural characteristics, a country where social engagement with physical contact is a tradition. Considering that, it is essential to investigate how social distancing may impact Brazilian citizens’ mental health. Another possible source of stress to the population is the economic crisis. Due to the partial lockdowns, many people lost their jobs (Dang et al., 2020), and, in the specific case of Brazil, the government provided a monthly aid of only 600 reais (approximately 100 dollars), which is far lower than the current minimum wage.

The present study’s objective was to investigate the pandemic’s impact on mental health and identify variables that can increase or decrease the chances of stress, anxiety, and depression, in both a sample with and one without self-reported mental health issues, in a Brazilian population.

## Design

### Participants

#### Sample size calculation

In order to obtain adequate statistical power (80%) to test the hypothesis of comparisons between two groups, considering a possible Cohen's effect size of *d *= 0.40, it would be necessary to obtain at least 99 participants for each group (practicing social distance and not practicing social distance). However, considering that to conduct multivariate regressions models, it is recommended at least 100 participants for each independent variable inserted in the model, we aimed to assess at least 1000 participants to enable the conduct of multivariate models appropriately.

Inclusion criteria were older than 18 years old (two volunteers were not included due to this criterion) and Brazilian Portuguese speakers.

### Procedures

The research had a quantitative cross-sectional character, and data were collected online in May and June of 2020. Social distancing measures started in Brazil in March. So, in the time of data collection, the participants had a mean of 60 days of social distancing. People were invited to participate in the research by using the non-probabilistic sample method ‘snowball.’ The disclosure was made on social networks by sending emails, phone messages, and the universities’ websites involved in the research. On the initial page of the form, the participants had access to the Free and Informed Consent Form (ICF), where they should select the option to accept the research, stating his awareness of the objectives and ethical issues involved to voluntary and anonymous participation. After the acceptance, access to the RedCap platform with questions to answer was released.

The National Ethics Committee approved the research under the Sentence number CAAE: 3 30966420.7.0000.5556

## Main outcome measures

*Socio-Demographic Questionnaire:* The instrument had 16 questions that sought to investigate the social and demographic profile of the participants, such as age, sex, education, marital status, profession, income, professional status identifying who has a stable bond or not (like the public agent, self-declared by the participant), the city where they live, as well as questions on the previous diagnosis of psychiatric diseases and if the participant has any diagnostic of the considered risk factors for worsening of COVID's condition (hypertension, diabetes, asthma, cancer, obesity, chronic kidney disease, immunological disease). Regarding the psychiatric diagnosis, it was asked if the person has any psychiatric diagnosis provided by a health professional with the option to mark yes or no and specify which diagnosis.

*Questionnaire to characterize social distance and the impact of social isolation on mental health:* The researchers developed the form and included 11 self-referenced questions answered by the participants. The social distance, questions were developed to identify whether the participant had been practicing social distance, if so, for how long, followed by questions that sought to assess the level of distancing through questions such as: leaves home only in case of real need or visits the home of family and friends eventually, thus differentiating a more rigid or less rigid social distance. It was also assessed if the participant increased the use of tranquilizers during the social distance period. To assess social support, participants were asked about the existence of a family and social support network (i.e. ‘Do you have relatives or friends you can count on, even if they are physically distant?’)

Depression, Anxiety, and Stress Scale – Short Form (DASS-21**)** (Lovibond & Lovibond, 1995): The DASS-21 consists of three subscales (Depression, Anxiety, and Stress), with seven items each. The participant must indicate on a four-point Likert scale (‘does not apply’ to ‘applies a lot, or most of the time’) or how much he/she experiences the symptom in the previous week. The scores should be separately computed for each subscale and range from 0 to 21. The instrument was adapted for Brazil (Vignola & Tucci, 2014), and after the translation and cross-cultural adaptation procedures, DASS-21 was applied to adults aged 18–75 years, finding Cronbach alphas ranging from 0.86–0.92 and following the three-factor structure (Depression, Anxiety, and Stress). DASS-21 has been used in previous COVID-19 studies (Tan et al., 2020; Tee et al., 2020). According to the DASS-21 cut-offs, participants were classified as very severe, severe, moderate, mild, or without symptoms of depression, anxiety, and stress. The cut-offs points for each category were as follows: without stress symptoms (0–14 pts), mild stress (15–16 pts), moderate stress (18–25 pts), severe stress (26–33 pts), very severe stress (above 34 pts); without anxiety symptoms (0–7pts), mild anxiety (8–9 pts), moderate anxiety (10–14 pts), severe anxiety (15–19 pts), very severe anxiety (above 20 pts); without depression symptoms (0–9 pts), mild depression (10–13 pts), moderate depression (14–20 pts), severe depression (21–27 pts), very severe depression (above 28pts) (Vignola & Tucci, 2014).

*Ways of Coping Questionnaire by* Folkman and Lazarus (1985): It is a questionnaire that contains 66 items that include thoughts and actions used by people to deal with internal or external demands of a specific stressful event. For this research, participants were asked to indicate the strategies they used to cope with the pandemia. The questionnaire is a Likert type 4 point scale. The four points might be indicating the frequency of each strategy is used: 0 to ‘does not apply/not used’, 1 to ‘used somewhat’, 2 to ‘used quite a bit’, and 3 to ‘used a great deal’. The version used was adapted and validated for the Brazilian population, with satisfactory psychometric properties (Savóia, Santana, & Mejias, 1996). The instrument has eight factors: positive reappraisal, social support, self-controlling, problem-solving, confrontation, distancing, accepting responsibility, and escape/avoidance. The meaning of each factor can be summarized as: Problem-solving: try to act within the possibilities, plan the actions, avoid failures, and do what is necessary to solve the problem and change de situation; Positive reappraisal – try to perceive a situation more positively, and try to get something positive out of it all, especially when it is not possible to completely solve the problem; Confrontation: it means that the person deals with the problem by looking for solutions and at the same time explaining his negative emotions; escape/avoidance: to feel better the person avoids the problem and prefers to live as if it did not exist; Distancing: the person seeks to move away so as not to feel emotionally overwhelmed, avoid thinking and worrying about this problem; Self-controlling: seeks to control emotions and have positive thoughts to feel more balanced; Social Support: the person counts on the nearby (family and friends) to support them; Acceptance of responsibility: individual tries to do its part because it understands that it is also part of the problem.

### Data analysis

Descriptive analyses were performed of socioeconomic, gender, anxiety, depression and stress levels, marital status, schooling, the prevalence of a previous mental disorder diagnostic, and practice of social distancing (if the participant was leaving home only in cases of extreme need or not leaving home, in comparison with those who are not practicing social distancing).

Categorical variables were individually associated with depression, anxiety, and stress classification, using the chi-square test. According to the Kolmogorov–Smirnov test, stress, anxiety, depression levels, and coping factors did not present normal distribution. Kruskal–Wallis tests were conducted to associate the quantitative variables with stress, anxiety, and depression classification.

Multinomial logistic regression models were conducted to identify the explaining models to moderate and severe depression, anxiety, and stress and investigate which variables present higher predictive value for mental health. Separate regressions were conducted considering as a dependent variable the presence of depression, anxiety, and stress separated into three groups: (1) very severe or severe symptoms; (2) moderate symptoms, and (3) mild or no symptoms, considering the latter as a reference for comparison with the other groups.

Univariate regressions were initially conducted for the variables that were significant in the bivariate associations. All the variables that were statistically significant in the univariate analysis were included in the multivariate regression models, adjusting the model for social income (income was considered as a confounding variable and because of this it was inserted in models using the Enter method, so the income influence was controlled).

To verify if there was any difference between the explaining model for those with or without any mental disorder diagnostic, we conducted two multinomial logistic regressions for each dependent variable (anxiety, depression, and stress): (a) regression for those who reported no mental health diagnostic; (b) regression for those who reported a previous diagnosis of mental health disorder. The analysis was conducted using the SPSS (Statistical Package for Social Sciences) for Windows version 22.00.

## Results

The research has 1316 individuals who answered the survey. However, 186 questionnaires presented missing data of DASS-21 or Way of Coping Questionnaire and, because of this, were excluded from the analysis. Participated in the study 1130 volunteers from 18 to 78 years old (Mean = 37.46 years; SD = 12.18), from 20 Brazilian states, 77.8% of whom were female.

### Sociodemographic information

Initially, the results show the descriptive analyses exploring more information about the participants. Participants varied regarding sociodemographic gender, monthly income, schooling, marital status, practicing social distancing, self-reported mental health diagnostic, and levels of anxiety, stress, and depression, as can be seen in (Table 1).

**Table 1.** Sample Descriptions and Prevalence of Depression, Anxiety and Stress symptoms.

Variables		*n*	(%)	X^2^
Gender	Male	250	22.1	
	Female	880	77.9	*p* < 0.01*
Income	<$580.00	303	27.0	
	$580.00 – $1,940.00	512	45.6	
	>$1,940.00	309	27.4	*p* < 0.01*
Schoolling	Elementary school	16	1.4	
	High School	98	8.7	
	University education	759	67.2	
	Master ou PhD degrees	257	22.7	*p* < 0.01*
Marital Status	Singles	427	39.4	
	Married or Stable Union	531	49.0	
	Divorced	111	10.2	
	Widowers	15	1.4	*p* < 0.01*
Practicing Social Distancing	Yes	778	70.5	
	No	326	29.5	*p* < 0.01*
Stable Bond	Yes	302	26.7	
	No	828	73.3	*p* < 0.01*
Family Support (My family cares about me and helps me when necessary)	Yes	783	69.3	
	No	347	30.7	*p* < 0.01*
Mental Health Diagnostic Reported	Yes	456	34.7	
	No	858	65.3	*p* < 0.01*
Depression Prevalence	Mild Symptoms	125	12,5	
	Moderate Symptoms	194	19.3	
	Severe or very severe Symptoms	173	17.2	*p* < 0.01*
Anxiety Prevalence	Mild Symptoms	84	8.3	
	Moderate Symptoms	143	14.1	
	Severe or very severe Symptoms			
		214	21.1	*p* < 0.01*
Stress Prevalence	Mild Symptoms	126	12.9	
	Moderate Symptoms	174	17.8	
	Severe or very severe Symptoms			
		187	19.2	*p* < 0.01*

* Chi-Square Test.

The profile of social distancing is characterized by 70.5% of the participants, who stated that they were practicing social distancing as a preventive measure against COVID-19, reporting that they are going out only in extreme need cases, such as going to market or drugstore. The mean time of social distancing was 58.61 days (*sd* = 23.2).

A significantly higher prevalence of severe depression was identified in those who were practicing social distancing (19.2% of participants in the social distance against 13.2% of those who were not in social distancing; *X²* = 8.45; *p *= 0.035). No significant differences were found between participants who were or not practicing social distancing regarding anxiety and stress. The time of social distancing was similar between participants and was not associated with depression, anxiety, and stress (*Kruskal–Wallis Test*; Depression *p* = 0.26; Anxiety *p* = 0.37; Stress *p* = 0.13).

Depression, anxiety, and stress were significantly higher in single people, followed by divorced and widowers, compared to married ones (Depression, *X²* = 47.6; *p* < 0.001; Anxiety, X² = 10.55; *p* = 0.03; Stress, X² = 22.4*, p* < 0.0001). Mean age was significantly higher in participants with no symptoms or mild symptoms of depression, anxiety and stress, in comparison with mean age of participants with moderate or severe levels of depression, anxiety and stress (Table 2).

**Table 2.** Description of the coping results (mean and standard deviation) separately by stress, depression and anxiety levels presented (DASS-21).

	Level	Age	Confrontation	Problem Solving	Escape	Positive Reappraisal	Distancing	Self-Control	Social Support	Accepting responsibility
*Participants without a mental health diagnostic*										
Depression	Mild/No symptoms	39.27 (11.38)	2.86 (2.66)	5.05 (3.00)	2.67 (1.86)	11.04 (5.66)	5.43 (3.44)	5.75 (2.71)	5.97 (3.29)	5.98 (4.08)
	Moderate	34.10 (11.07)	4.97 (2.70)	4.11 (2.34)	4.10 (1.90)	9.94 (5.41)	7.03 (3.24)	6.6.1 (2.72)	6.73 (2.87)	7.65 (3.84)
	Severe	29.78 (10.24)	6.96 (5.72)	3.59 (2.42)	4.61 (1.89)	8.58 (5.38)	8.41 (3.94)	6.54 (2.71)	6.27 (3.69)	8.22 (3.99)
	Kruskal-Wallis (*p*)	<0.001**	<0.001**	<0.001**	<0.001**	<0.001**	<0.001**	<0.001**	0.105	<0.001**
Anxiety	Mild/No symptoms	38.45 (11.69)	2.92 (2.57)	4.79 (3.00)	2.76 (1.95)	10.36 (5.67)	5.52 (3.47)	5.77 (2.83)	5.83 (3.23)	5.94 (3.97)
	Moderate	36.08 (13.02)	4.91 (3.23)	4.52 (2.58)	4.08 (1.76)	10.93 (5.53)	7.35 (3.60)	6.46 (2.58)	6.60 (3.00)	8.32 (3.90)
	Severe	32.04 (10.14)	7.26 (5.94)	4.39 (2.55)	4.44 (1.85)	10.84 (5.76)	7.82 (3.60)	6.90 (2.58)	7.28 (3.59)	8.22 (4.16)
	Kruskal-Wallis (*p*)	<0.001**	<0.001**	0.491	<0.001**	0.898	<0.001**	<0.001**	0.004**	<0.001**
Stress	Mild/No symptoms	39.21 (11.79)	2.84 (2.80)	5.00 (2.95)	2.67 (1.81)	10.82 (5.63)	5.60 (3.37)	5.85 (2.79)	5.82 (3.14)	5.98 (3.96)
	Moderate	33.72 (9.96)	5.08 (2.97)	4.31 (2.64)	3.91 (1.78)	11.40 (5.40)	7.25 (3.57)	6.40 (2.70)	7.01 (3.30)	7.82 (3.95)
	Severe	31.20 (10.59)	6.60 (3.35)	4.30 (2.50)	4.48 (1.55)	9.70 (5.48)	8.14 (3.90)	6.81 (2.62)	7.04 (3.60)	6.60 (3.35)
	Kruskal-Wallis (*p*)	<0.001**	<0.001**	0.03*	<0.001**	0.059	<0.001**	0.002**	0.001**	<0.001**
*Participants with a mental health diagnostic*										
Depression	Mild/No symptoms	40.99 (12.74)	3.66 (2.66)	4.61 (2.37)	2.91 (2.09)	10.51 (5.29)	5.70 (3.48)	5.26 (2.60)	6.96 (3.66)	5.97 (4.21)
	Moderate	33.57 (9.97)	5.69 (3.27)	4.16 (2.48)	4.32 (1.84)	9.98 (5.84)	7.30 (2.97)	6.12 (2.39)	7.21 (3.48)	7.29 (3.55)
	Severe	33.04 (10.75)	6.82 (3.60)	3.85 (2.61)	4.72 (2.08)	9.63 (5.27)	8.08 (3.61)	6.95 (2.65)	7.17 (3.27)	9.60 (4.25)
	Kruskal-Wallis (*p*)	<0.001**	<0.001**	0.093	<0.001**	0.229	<0.001**	<0.001**	0.729	<0.001**
Anxiety	Mild/No symptoms	40.87 (12.70)	3.40 (2.98)	4.35 (2.66)	2.92 (2.11)	9.70 (5.33)	5.84 (3.40)	5.32 (2.55)	6.09 (3.28)	5.71 (4.05)
	Moderate	35.51 (12.28)	5.40 (3.11)	4.19 (2.48)	3.35 (1.99)	10.76 (5.44)	6.89 (3.27)	5.73 (2.38)	8.00 (3.92)	7.70 (3.83)
	Severe	33.59 (10.43)	6.61 (3.24)	4.25 (2.34)	4.89 (1.86)	10.19 (5.52)	7.76 (3.62)	6.86 (2.60)	7.67 (3.18)	8.95 (4.28)
	Kruskal-Wallis (*p*)	<0.001**	<0.001**	0.899	<0.001**	0.769	0.006**	<0.001**	0.001**	<0.001**
Stress	Mild/No symptoms	41.15 (13.12)	3.37 (2.38)	4.39 (2.43)	2.94 (1.96)	10.02 (5.59)	5.75 (3.29)	5.22 (2.66)	6.38 (3.79)	5.93 (3.98)
	Moderate	34.31 (9.74)	5.05 (3.31)	4.16 (2.45)	3.43 (1.76)	10.56 (4.83)	7.24 (2.72)	6.49 (2.32)	7.43 (2.85)	7.76 (4.14)
	Severe	34.08 (10.53)	6.99 (3.01)	4.19 (2.60)	4.60 (1.75)	10.25 (5.31)	7.90 (3.74)	6.46 (2.52)	7.76 (3.35)	8.87 (4.40)
	Kruskal-Wallis (*p*)	<0.001**	<0.001**	0.941	<0.001**	0.972	0.001**	0.003**	0.012**	<0.001**

Being in the considered risk groups for worsening of COVID's condition was not associated with higher levels of depression, anxiety, and stress (Depression, *X²* = 3.34, *p* = 0.10; Anxiety, *X²* = 4.57, *p *= 0.10; Stress, *X² =* 2.44, *p *= 0.29). Informal workers presented higher anxiety levels *(p* = 0.01), and the unemployed presented higher depression levels (*p* = 0.02) than the other participants. Family support was significantly associated with mental health since participants with severe depression and stress reported that they did not receive support from family (Depression, *X²* = 20.88, *p* < 0.001; Anxiety, *X²* = 11.93, *p* = 0.03; Stress *X²* = 16.55, *p* < 0.001).

34,7% of participants reported a previous diagnostic of mental health, being 14.6% anxiety and 6.4% depression. Considering this result, we conducted separate analyses for these groups to investigate if there is any difference between the coping strategies used by those with mental disorders in comparison with those who reported no mental diagnostic. There are no significant differences regarding gender, age, or social income (*p *> 0.05) on participants with or without a mental diagnosis.

### Coping strategies

We investigated the coping strategies used by participants to face social distancing and pandemic fear. The factors confrontation, escape, distancing, self-controlling and accepting responsibility were significantly more used by participants that presented the highest levels of stress, anxiety, and depression, for both participants with and without a previous mental health diagnostic (*Kruskal–Wallis* Test, *p* < 0.05, Table 2). The problem-solving strategy was more used by participants with lower levels of depression (*Kruskal–Wallis Test, p* < 0.0001) and stress (*Kruskal–Wallis Test, p* = 0.03), but only for participants without a previous mental health diagnostic, suggesting that it can be an efficient coping strategy. The positive reappraisal was a strategy more used by participants with lower levels of depression, for the participants without a previous mental health diagnostic (*Kruskal–Wallis Test, p* < 0.0001) (Table 2).

### Regression models

Multinomial logistic regression models were conducted to identify the explaining models to moderate and severe depression, anxiety, and stress. The logistic regression result found explaining models of risk and protective variables to depression, anxiety, and stress. The results were split into two parts; first, we described the results of people who did not declare mental disorders, and, in sequence, we described the results of people who declared they were diagnosed with mental disorders.

### Depression in participants without mental health diagnostic

#### Moderate depression

Initially, we conducted univariate regressions considering as independent variables those that were significant in the bivariate association with depression (age, marital status, stable bond, family support, the practice of social distancing, and coping strategies). In the multivariate regression, income was considered as a confounding variable and inserted in the model using the Enter method in order to adjust the variables to the possible income effects.

Considering the group without any mental disorder diagnosis, the variables age (lower age), marital status (singles and widowers), stable bond, coping strategies distancing, self-controlling, and accepting responsibility were significant risk factors for moderate depression (Table 3). However, in the multivariate models, these variables lost significance, and only the practice of social distancing *(OR* = 1.85, *IC95%*1.05–3.26), and coping strategies confrontation (*OR *= 1.24, *IC95%* 1.12–1.37), escape (*OR* = 1.44, *IC95%* 1.22–1.69) and distancing (*OR *= 1.09, *IC95%* 1.00–1.20) were identified as variables that increase the risk of moderate depression. The coping strategies of problem-solving (*OR* = 0.81, *IC95%* 0.72–0.92) and positive reappraisal (*OR* = 0.87, *IC95%* 0.82–0.94) presented a protective effect, decreasing the chances of moderate depression (Table 3), for participants without a previous mental health diagnostic.

**Table 3.** Multinomial Logistic Regression Model, Stepwise Method adjusted for income, for Dependent Variable Depression (Severe and Moderate Depression in comparison to mild or no Depression symptoms)

		Participants without mental health diagnostic						Participants with mental health diagnostic					
		Univariate			Multivariate Model			Univariate Analyzes			Multivariate Model		
		OR	95% CI	*p*	OR adj	95% CI	*p*	OR	95% CI	*p*	OR adj	95% CI	*p*
*Moderate Depression*													
Age		0.96	0.94-0.97	<0.001	1.01	0.98-1.04	0.44	0.95	0.92-0.98	<0.001	0.95	0.90-1.00	0.072
Social Distancing	Yes	1.20	0.78–1.83	0.40	1.85	1.05-3.26	0.03	2.62	1.18–5.8	0.018	4.26	1.47-12.36	0.008
Marital Status (Reference: married)	Singles	2.36	1.17–4.77	0.01	1.63	0.62-4.31	0.32	2.27	0.67–7.72	0.19	0.57	0.09–3.42	0.537
	Divorced or Widower	1.17	0.58–2.35	0.66	0.97	0.41-2.30	0.94	1.59	0.47–5.33	0.45	1.61	0.34–7.74	0.550
Stable Bond		0.56	0.37–0.88	0.01	0.69	0.38-1.27	0.24	0.83	0.42–1.64	0.58	2.12	0.73–6.16	0.169
Family Support	Do not have	1.44	0.88–2.34	0.14	1.00	0.48-2.11	0.99	1.13	0.53–2.40	0.76	1.72	0.53–5.61	0.368
Confrontation		1.80	1.19–1.37	<0.001	1.24	1.12-1.37	<0.001	1.26	1.23–1.56	<0.001	1.26	1.04–1.53	0.017
Problem solving		0.88	0.75–0.89	0.001	0.81	0.72-0.92	0.001	0.91	0.79–1.04	0.176	0.96	0.77–1.19	0.705
Escape		1.48	1.32–1.65	<0.001	1.44	1.22-1.69	<0.001	1.41	1.18–1.69	<0.001	1.44	1.09–1.88	0.008
Positive Reappraisal		0.96	0.93–0.99	0.02	0.87	0.82-0.94	<0.001	0.98	0.92–1.04	0.44	0.98	0.87–1.11	0.749
Distancing		1.14	1.08–1.2	<0.002	1.09	1.00-1.20	0.042	1.15	1.03–1.28	0.009	1.03	0.86–1.24	0.722
Self-Control		1.12	1.04–1.20	0.002	1.04	0.93-1.17	0.498	1.15	0.99–1.32	0.052	1.03	0.83–1.28	0.790
Social Support		1.06	0.99–1.13	0.06	1.02	0.91-1.13	0.775	1.03	0.93–1.13	0.61	0.97	0.82–1.13	0.663
Accepting responsibility		1.10	1.05–1.16	<0.001	1.05	0.96-1.16	0.284	1.07	0.98–1.17	0.10	0.96	0.82–1.13	0.650
*Severe Depression*													
Age		0.92	0.89-0.94	<0.001	0.99	0.95-1.03	0.71	0.94	0.92-0.97	<0.001	0.95	0.89–1.02	0.132
Social Distancing	Yes	1.67	0.98–2.84	0.056	2.37	1.10-5.08	0.027	2.00	1.03–3.87	0.04	3.19	1.11–9.18	0.032
Marital Status (Reference: married)	Singles	3.15	1.43–6.95	0.004	2.32	0.60-8.95	0.221	1.25	0.49–3.18	0.64	0.48	0.07–3.30	0.452
	Divorced or Widower	0.49	0.20–1.18	0.11	0.38	0.10-1.41	0.15	0.84	0.33–2.1	0.71	1.59	0.30–8.35	0.583
Stable Bond		0.49	0.29–0.83	0.008	0.78	0.32–1.93	0.60	0.61	0.32–1.16	0.13	1.53	0.48–4.91	0.472
Family Support	Do not have	2.70	1.64–4.43	<0.001	2.57	1.08–6.13	0.033	1.53	0.78–2.97	0.21	1.95	0.57–6.63	0.286
Confrontation		1.47	1.33–1.55	<0.001	1.39	1.23–1.58	<0.001	1.39	1.23–1.56	<0.001	1.33	1.10–1.60	0.004
Problem solving		0.82	0.75–0.89	<0.001	0.75	0.63–0.88	0.001	0.88	0.77–0.99	0.046	0.93	0.73–1.18	0.541
Escape		1.67	1.47–1.93	<0.001	1.48	1.19–1.84	<0.001	1.52	1.28–1.81	<0.001	1.49	1.12–1.98	0.007
Positive Reappraisal		0.91	0.87–0.95	<0.001	0.85	0.78–0.93	0.001	0.96	0.91–1.02	0.22	0.92	0.80–`0.05	0.196
Distancing		1.24	1.16–1.32	<0.001	1.20	1.07–1.35	0.002	1.22	1.10–1.35	<0.001	0.96	0.79–1.16	0.653
Self – Control		1.13	1.04–1.23	0.005	0.99	0.86–1.17	0.960	1.35	1.17–1.54	<0.001	1.11	0.89–1.39	0.349
Social Support		1.01	0.95–1.09	0.68	0.95	0.83–1.09	0.450	1.03	0.94–1.13	0.55	0.92	0.78–1.09	0.350
Accepting responsibility		1.12	1.06–1.19	<0.001	1.08	0.96–1.23	0.200	1.22	1.12–1.33	<0.001	1.17	0.99–1.38	0.052

#### Severe depression

The predictors for the level of severe depression were similar to those for moderate depression, with the practice of social distancing (*OR* = 2.37; *IC95%* 1.10–5.08) and coping factors confrontation (*OR* = 1.39; *IC95%* 1.23–1.58), escape (*OR* = 1.48; *IC95% *= 1.19–1.84) and distancing (*OR* = 1.20; *IC95%* 1.07–1.35) increasing the risk of severe depression, and the problem solving (*OR* = *0.75; IC95*% 0.63–0.88) as also positive reappraisal (*OR* = *0.85; IC95*% 0.78–0.93) were strategy that showed significance in the model to reduce the chance of severe depression (Table 3). The absence of family support was also a significant risk factor for severe depression (*OR* = *2.57; IC95*% 1.08–6.13).

### Depression in participants that reported previous mental health diagnostic

#### Moderate depression

Risk and protection factors associated with depression were also analyzed in individuals who suffer from mental disorders. The predictors that increase the odds of moderate depression were similar to those for participants without a previous mental disorder: practice of social distancing (*OR* = 4.26; *IC95%* 1.47–12.36) and coping factors confrontation (*OR* = 1.26; *IC95%* 1.04–1.53), escape (*OR* = 1.44; *IC95%* 1.09–1.88). No significant predictors were associated to reduce the odds of moderate depression for this group (Table 3).

#### Severe depression

The predictors for the level of severe depression were similar to those for moderate depression, with the practice of social distancing (*OR* = 3.19; *IC95%* 1.11–9.18) and coping factors confrontation (*OR* = 1.33; *IC95%* 1.10–1.60) and escape (*OR* = 1.49; *IC95%* 1.12–1.98) as risk factors for severe depression for this group (Table 3).

### Anxiety in participants without mental health diagnostic

#### Moderate anxiety

Univariate multinomial regressions were conducted considering anxiety level as dependent variable (severe or moderate in comparison to mild or no symptoms) and age, practice social distancing, marital status, stable bond, social support, and coping strategies as independent variables.

For the group that reported not having any mental diagnostic, individuals with moderate anxiety are more prone to using escape *(OR* = 1.27, *IC95%* 1.06–1.51) and accepting responsibility strategies *(OR* = 1.12, *IC95%* 1.02–1.26). Age was also a significant risk factor, with a small value of odds ratio (*OR* = 1.03, *IC95%* 1.00–1.07). Not having family support increased in 2.12 times the odds of presenting moderate anxiety *(OR* = 2.12, *IC95%* 1.05–4.31). Problem solving strategy reduced the odds of moderate anxiety *(OR* = 0.86, *IC95%* 0.75–0.98) (Table 4).

**Table 4.** Multinomial Logistic Regression Model, Stepwise Method adjusted for income, for Dependent Variable Anxiety (Severe and Moderate Anxiety in comparison to mild or no Anxiety symptoms)

		Participants without mental health diagnostic						Participants with mental health diagnostic					
		Univariate			Multivariate Model			Univariate Analyzes			Multivariate Model		
		OR	95% CI	*p*	OR adj	95% CI	*p*	OR	95% CI	*p*	ORadj	95% CI	*P*
*Moderate Anxiety*													
Age		0.98	0.96–1.00	0.07	1.04	1.00–1.07	0.02	0.96	0.93–0.99	0.91	0.12	0.90–1.01	0.12
Social Distancing	Yes	0.96	0.601–1.55	0.88	0.86	0.48–1.65	0.62	0.75	0.35–1.61	0.46	1.77	0.62–5.03	0.28
Marital Status (Reference: married)	Singles	1.89	0.81–4.43	0.141	2.27	0.74–6.90	0.14	1.43	0.43–4.67	0.55	0.68	0.11–4.31	0.68
	Divorced or Widower	1.71	0.74–3.94	0.21	2.41	0.99–6.51	0.08	1.03	0.32–3.29	0.96	1.68	0.29–8.57	0.58
Stable Bond		0.62	0.38–1.03	0.06	0.74	0.38–1.42	0.36	0.55	0.25–1.21	0.14	0.12	0.35–3.56	0.85
Family Support	Do not have	1.54	0.90–2.64	0.11	2.12	1.05–4.31	0.04	0.47	0.18–1.25	0.13	0.16	0.03–1.00	0.05
Confrontation		1.28	1.18–1.39	<0.001	1.12	1.00–1.26	0.05	1.30	1.14–1.49	<0.001	1.29	1.10–1.56	0.01
Problem solving		0.96	0.89–1.04	0.30	0.86	0.75–0.98	0.02	0.98	0.84–1.13	0.76	0.84	0.66–1.07	0.16
Escape		1.43	1.26–1.62	<0.001	1.27	1.06–1.51	0.009	1.18	0.97–1.43	0.10	0.82	0.61–1.10	0.18
Positive Reappraisal		1.00	0.96–1.04	0.87	0.98	0.91–1.05	0.58	1.03	0.96–1.10	0.44	1.05	0.93 −1.19	0.43
Distancing		1.14	1.07–1.21	<0.001	0.86	0.48–1.65	0.62	1.10	0.98–1.23	0.10	0.92	0.83–1.21	0.92
Self-Control		1.08	0.99–1.17	0.09	0.93	0.82 −1.06	0.28	1.06	0.91–1.24	0.45	0.93	0.72–1.19	0.56
Social Support		1.06	0.99–1.13	0.119	1.01	0.90–1.14	0.81	1.18	1.05–1.32	0.006	1.28	1.08–1.51	0.005
Accepting responsibility		1.14	1.08–1.20	<0.001	1.12	1.02 −1.26	0.02	1.14	1.03–1.26	0.008	1.00	0.85–1.19	0.96
*Severe Anxiety*													
Age		0.94	0.93–0.96	<0.001	0.98	0.95–1.01	0.28	0.95	0.92–0.97	<0.001	0.96	0.90–1.04	0.10
Social Distancing	Yes	1.21	0.77–1.90	0.41	1.79	0.96–3.35	0.06	1.67	0.85–3.23	0.14	3.34	1.27–9.58	0.02
Marital Status (Reference: married)	Singles	2.00	0.97–4.14	0.06	1.95	0.58–6.53	0.28	1.71	0.64–4.55	0.28	0.81	0.14–4.56	0.81
	Divorced or Widower	1.20	0.57–2.49	0.63	1.97	0.64–6.00	0.23	1.16	0.43–2.91	0.82	2.04	0.44–9.41	0.36
Stable Bond		0.65	0.42–1.03	0.06	0.96	0.48–1.91	0.90	0.78	0.43–1.42	0.41	1.90	0.65–5.58	0.24
Family Support	Do not have	1.73	1.07–2.81	0.02	1.68	0.80–3.55	0.17	1.43	0.752–2.71	0.27	1.97	0.66–5.91	0.22
Confrontation		1.47	1.35–1.58	<0.001	1.38	1.24–1.54	0.000	1.43	1.26–1.61	<0.001	1.28	1.08–1.53	0.005
Problem solving		0.95	0.88–1.02	0.18	0.82	0.72–0.93	0.03	1.00	0.89–1.12	0.99	0.96	0.78–1.18	0.70
Escape		1.59	1.40–1.79	<0.001	1.34	1.12 −1.60	0.01	1.73	1.43–1.08	<0.001	1.52	1.16–1.99	0.003
Positive Reappraisal		1.09	0.97–1.05	0.63	0.81	0.94–1.08	0.92	1.02	0.97–1.08	0.48	0.96	0.86–1.07	0.48
Distancing		1.19	1.12–1.26	<0.001	1.01	0.91–1.11	0.81	1.18	1.07–1.29	0.001	0.95	0.30–1.13	0.53
Self – Control		1.16	1.07–1.26	<0.001	1.01	0.89–1.15	0.84	1.30	1.14–1.48	<0.001	1.06	0.86–1.29	0.59
Social Support		1.11	1.04–1.18	0.001	1.07	0.95–1.20	0.24	1.16	1.06–1.28	0.002	1.12	0.96–1.31	0.14
Accepting responsibility		1.35	1.08–1.19	<0.001	0.96	0.86–1.05	0.38	1.22	1.12–1.32	<0.001	1.07	0.93–1.23	0.35

#### Severe anxiety

Confrontation (*OR* = 1.38, I*C95%* 1.24–1.54) and escape *(OR* = 1.34, *IC95%* 1.12–1.60) were risk factors for severe anxiety. Problem solving was also a protective factor for severe anxiety (*OR* = 0.82, *IC95%* 0.72–0.93) (Table 4).

### Anxiety in participants that reported previous mental health diagnostic

#### Moderate anxiety

In the multivariate model, the significant variable that explained moderate anxiety for those who reported previous mental health diagnoses was confrontation (*OR* = 1.46, *IC95%* 1.03–2.06) (Table 4), meaning that participants with moderate anxiety presented a higher chance of using the confrontation strategy.

#### Severe anxiety

Practice of social distancing increased in 3.48 times the odds of severe depression in participants with previous mental disorders *(OR* = 3.48, *IC95%* 1.27–9.58). Coping strategies confrontation (*OR* = 1.28, *IC95%* 1.08–1.53) and escape (*OR* = 1.52, *IC95%* 1.16–1.99) were also risk factors for severe anxiety for this group. No variables with a protective effect for severe anxiety were found for this group (Table 4).

### Stress in participants without mental health diagnostic

#### Moderate stress

Considering the group without any mental disorder diagnosis, confrontation (*OR* = 1.29, *IC95%* 1.15–1.44) and escape (*OR* = 1.24, *IC95%* 1.06–1.46) strategies increases the odds to moderate stress (Table 5). Problem solving (*OR* = *0.81*, *IC95%* 0.72–0.92) was a protective factor for moderate stress.

**Table 5.** Multinomial Logistic Regression Model, Stepwise Method adjusted for income, for Dependent Variable Stress (Severe and Moderate Stress in comparison to mild or no Stress symptoms).

		Participants without mental health diagnostic						Participants with mental health diagnostic					
		Univariate			Multivariate Model			Univariate Analyzes			Multivariate Model		
		OR	95% CI	*p*	OR adj	95% CI	*P*	OR	95% CI	*p*	OR adj	95% CI	*p*
*Moderate Stress*													
Age		0.95	0.94–0.97	<0.000	0.97	0.94–1.00	0.08	0.95	0.92–0.98	0.003	0.94	0.88–0.99	0.28
Social Distancing	Yes	0.71	0.47–1.06	0.09	0.75	0.44–1.29	0.29	0.84	0.39–1.82	0.67	1.56	0.55–4.43	0.41
Marital Status (Reference: married)	Singles	2.24	1.04–4.80	0.04	1.18	0.40–3.51	0.77	1.43	0.43–4.67	0.55	0.73	0.12–4.36	0.23
	Divorced or Widower	1.87	0.89–3.94	0.10	1.34	0.51–3.55	0.55	1.03	0.32–3.29	0.96	0.27	0.25–6.47	0.77
Stable Bond		0.69	0.45–1.06	0.09	0.81	0.43–1.51	0.50	0.55	0.25–1.21	0.14	2.52	0.82 – 7.74	0.11
Family Support	Do not have	1.44	0.88–2.34	0.14	1.30	0.60–2.80	0.51	0.47	0.18–1.25	0.13	2.79	0.79–9.88	0.11
Confrontation		1.36	1.26–1.47	<0.001	1.29	1.15–1.44	0.00	1.35	1.16–1.58	<0.001	1.37	1.10–1.71	0.05
Problem solving		0.91	0.84–0.99	0.02	0.81	0.72–0.92	0.001	0.98	0.86–1.13	0.84	0.85	0.67–1.08	0.17
Escape		1.46	1.29–1.65	<0.001	1.24	1.06 −1.46	0.009	1.16	0.96–1.41	0.11	0.95	0.71–1.25	0.67
Positive Reappraisal		1.02	0.98–1.05	0.28	1.00	0.93–1.07	0.96	1.00	0.93–1.07	0.94	1.00	0.88–1.13	0.98
Distancing		1.13	1.06–1.2	<0.001	0.30	0.96–1.15	0.29	1.14	1.01–1.28	0.03	1.56	0.55–4.43	0,41
Self-Control		1.07	0.99–1.15	0.08	0.95	0.84–1.07	0.35	1.19	1.03–1.39	0.01	1.11	0.89–1.40	0.36
Social Support		1.11	1.05–1.19	<0.001	1.06	0.95–1.18	0.30	1.10	0.98–1.23	0.08	1.12	0.95–1.32	0.19
Accepting responsibility		1.11	1.05–1.16	<0.001	1.01	0.92–1.11	0.86	1.12	1.02–1.22	0.02	0.98	0.83–1.14	0.76
*Severe Stress*													
Age		0.93	0.91–0.97	<0.001	0.99	0.95–1.02	0.43	0.95	0.92–0.97	<0.001	0.97	0.91–1.02	0.23
Social Distancing	Yes	0.85	0.53–1.36	0.51	1.05	0.55–2.00	0.89	1.02	0.57–2.03	0.83	1.69	0.61–4.61	0.32
Marital Status (Reference: married)	Singles	2.91	1.32–6.41	0.008	2.10	0.58–7.47	0.26	1.88	0.70–5.00	0.21	1.23	0.19–7.88	0.82
	Divorced or Widower	0.92	0.40–2.11	0.90	1.10	0.34–3.56	0.88	1.60	0.62–4.16	0.34	4.00	0.76–20.94	0.10
Stable Bond		0.65	0.39–1.06	0.08	1.01	0.48–2.12	0.98	0.60	0.61–2.66	0.50	0.74	0.25–0.2.23	0.59
Family Support	Do not have	2.70	1,64–4.43	<0.001	2.03	0.91–4.55	0.08	1.53	0.78–2.97	0.21	2.73	0.84–8.82	0.09
Confrontation		1.53	1.34–1.67	<0.001	1.45	1.29–1.64	<0.001	1.64	1.41–1.90	<0.001	1.64	1.33–2.00	0.00
Problem solving		0.91	0.89–0.99	0.03	0.88	0.77–1.01	0.08	0.98	0.86–1.09	0.69	0.94	0.76–1.17	0.59
Escape		1.79	1.55–2.08	<0.001	1.52	1.25–1.80	<0.001	1.68	1.38–2.02	<0.001	1.26	0.99–1.65	0.10
Positive Reappraisal		0.95	0.92–1.00	0.05	0.90	0.83–0.98	0.01	1.00	0.95–1.06	0.80	0.97	0.67–1.10	0.57
Distancing		1.20	1.13–1.28	<0.001	1.07	0.97–1.19	0.17	1.20	1.10– 1.33	<0.001	1.07	0.90–1.26	0.47
Self – Control		1.16	1.06–1.26	0.001	1.03	0.90–1.18	0.58	1.25	1.10–1.43	<0.001	1.00	0.87–1.24	0.97
Social Support		1.08	1.01–1.16	0.01	1.09	0.96–1.23	0.17	1.13	1.03–1.24	0.01	1.10	0.92–1.27	0.36
Accepting responsibility		1.14	1.07–1.20	<0.001	0.97	0.86–1.08	0.58	1.19	1.10–1.29	<0.001	0.96	0.83–1.12	0.60

#### Severe stress

Confrontation (*OR* = 1.45, *IC95%* 1.29–1.64) and escape strategies *(OR* = 1.52, *IC95%* 1.25–1.85) also increased the odds of severe stress for participants without a mental health diagnostic (Table 5). The use of positive reappraisal strategy reduced the odds of severe stress (*OR* = 0.90, *IC95%* 0.83–0.98)

### Stress in participants that reported previous mental health diagnostic

#### Moderate stress

For the group that reported a mental disorder diagnostic, confrontation strategy increased the odds of moderate stress (*OR* = 1.37, *IC95%* 1.10–1.71). Age was a protective factor, since higher age reduced the odds of moderate stress (*OR* = 0.94, *IC95%* 0.88–0.99) (Table 5).

#### Severe stress

Confrontation increases the odds of severe stress (*OR* = 1.64, *IC95%* 1.33–2.00) for participants with a mental health diagnostic. There were no significant variables in the model for reducing the severe stress in this group (Table 5).

In general, the practice of social distancing increased the odds of presenting depression and anxiety. The mean age was lower for higher levels of depression, anxiety and stress in the univariate regressions. Being single, divorced, or widowed increased the chance of presenting worse mental health, considering the univariate regressions. Otherwise, marital status and stable bond were not statistically significant predictors in the multivariate regressions for explaining depression, anxiety and stress levels. Social support was a significant explanatory factor for mental health, since the absence of a family support network increased in approximately 2.0 times the odds of presenting anxiety and depression in participants without a previous mental health diagnosis.

Coping strategies were significant explanatory variables to mental health in the multivariate models. Confrontation and escape strategies increased the odds of presenting higher levels of depression, stress and anxiety. Accepting responsibility was a strategy that increased the odds of moderate anxiety in participants without previous mental health disorders. Positive reappraisal and problem solving were protective factors, decreasing the odds of presenting higher levels of depression, anxiety and stress, but only for participants without a previous mental health diagnostic.

## Discussion

In this study, the objective was to investigate the pandemic’s impact on mental health and identify variables that can increase or decrease the chances of stress, anxiety, and depression, in both a sample with and one without self-reported mental health issues. To analyze the variables that increase the risk or have a protective effect on mental health, socio-demographic variables such as job stability, marital status, family support, and coping strategies were tested.

In summary, the main findings indicate rates between 15–20% of people with moderate or severe symptoms of stress, depression, and anxiety. A significantly higher prevalence of severe depression was identified in those who were practicing social distancing than those who were not. The youngest was more vulnerable to psychological disorders. Being single, divorced, or widowed increases the risk of depression. Escape, accepting responsibility, and confrontation were identified as coping strategies that worsen mental health rates during the pandemic. Problem-solving and positive reappraisal were protective coping strategies that potentially reduced the odds of presenting depression and anxiety, but only in people without a previous mental health diagnosis. These findings will be discussed in further details.

The levels of anxiety, depression, and stress were higher for younger individuals (around 30 years). Age was also significant in univariate regressions for the Anxiety, Depression, and Stress outcomes, with higher levels of symptoms for younger individuals. Similar results were found in China's studies (Huang & Zhao, 2021; Wang, Xia, Xiong, Li, Xiang, Yuan, et al., 2020) and in Portugal (Moreira et al., 2020). The highest vulnerability was identified for younger individuals. This effect demonstrates that the risk to mental health in young people may be associated with uncertainties regarding the economic situation and professional activities. It is essential to highlight that increasing age is a protective factor triggered by increased functional coping strategies and resilient behavior (Browne-Yung, Walker, & Luszcz, 2017).

The number of people with depression was significantly higher in participants practicing social distancing than those who were not. The discomfort caused by social distancing can be one of the causes of low adherence to this measure since the average adherence to social distancing in Brazil was around 50%, and the recommended is at least 70% of the population exercising social distance. On the other hand, it is necessary to consider that the number of people with mental disorders may significantly increase due to prolonged social distancing.

Social distancing, being single, divorced or widower were significant variables for the risk of mental disorders during the pandemic, suggesting that few social interactions may aggravate symptoms of depression, stress, and anxiety in the pandemia context. There is evidence that loneliness worsens the perception of well-being and quality of life (Lewis, 2016). The adverse effects of loneliness interfere with reduced immunity and increased stress and depression (Campagne, 2019). Research carried out in China on the pandemic's impacts on mental health also identified that the young and single population had a higher level of psychological distress (Wang, Xia, Xiong, Li, Xiang, Yuan, et al., 2020).

Researchers warn of the high risk of a psychiatric illness pandemic considering the countless causes triggered by the COVID-19 pandemic such as high level of insecurity, confusion, emotional isolation and economic losses, reduced activities, closing schools, few and inadequate mental health services (Pfefferbaum & North, 2020; Torales, O'Higgins, Castaldelli-Maia, & Ventriglio, 2020; Yao et al., 2020). In the analysis by Booker et al. (2020), some people manage to overcome the effects, but, for most people, events with a high magnitude of stress such as disasters and pandemics can trigger trauma and a significant number of psychopathologies.

Rajkumar (2020) corroborates this by indicating the presence of symptoms of anxiety and depression (16–28%), stress (8%) as common reactions to the harmful effects of the COVID-19 pandemic, including possible sleep disorders. The number of adults who fulfilled the criteria for mental disorders increased eightfold in the US, comparing the mental health data collected during April 2020 with similar data collected in 2018 (Twenge & Joiner, 2020).

Despite the effect found for the social distancing and marital status variables, the regression models demonstrated that social support was a factor of protection for mental health. Social support is a resource that considers the perception of the availability of family and friends to offer material or psychological support (Haber, Cohen, Lucas, & Baltes, 2007). Social support increases the possibilities of sharing feelings, feeling secure, and receiving comfort even for situations that do not have to be modified (Haber et al., 2007), as is the case of the pandemic. This resource has been identified as a positive predictor for reducing the pandemic's adverse effects (Xiao, Zhang, Kong, Li, & Yang, 2020; Zhang & Ma, 2020). Participants who reported that they do not have family support were more likely to experience depression and anxiety. This result was also found in other studies (El-Zoghby, Soltan, & Salama, 2020; Roy, 2020; Wu et al., 2020). The attitude of seeking support from family and friends during the pandemic showed a 63% reduction in symptoms of depression by 63% and a 52% less chance of sleep disorders (Grey et al., 2020). In this study, the authors argued that having family and friends available to offer material or psychological support is an important way of minimizing the emotional effects of the pandemic's social detachment. The consistency of the findings that indicate social support as a protective factor suggests that this is a crucial way to increase coping capacity and decrease depression and anxiety during the pandemic.

Regression models identified which coping strategies were significant predictors of risk or protection for stress, anxiety, and depression. The models’ main findings indicated that the confrontation strategy increased the odds of moderate and severe depression, anxiety, and stress. The confrontation strategy corresponds to an offensive way of dealing with situations. People who use this strategy deal with problems by looking for solutions and expressing their unpleasant emotions. Another strategy that was significant for increasing the risk was the escape factor, considered a risk for depression, anxiety and stress. The escape strategy is characterized by an effort to avoid the stressor, which reduces the stressor's discomfort (Taylor & Stanton, 2007). The escape strategy was also employed by Malaysia's population to deal with the adverse effects of COVID-19, with individuals avoiding to think about the problem, considering that nothing can be done to change the situation (Perveen, Hamzah, Ramlee, Othman, & Minhad, 2020). Both forms of coping, escape, and confrontation are part of strategies focused on emotion. The coping style focused on emotion reflects defensive and detachment processes from the problem, focusing its action on regulating or replacing the emotional impact of stress (Penley, Tomaka, & Wiebe, 2002).

Coping strategies focused on emotion are more used in stressful situations with few possibilities for immediate solutions to problems. A pandemic is a stressful event associated with high levels of stress, anxiety, and depression. However, the management to face the pandemic is not under the direct control of people. On the contrary, the primary way to decrease the number of cases is social distancing, which decreases interactions and increases loneliness.

Despite the escape and confrontation showing increased risk, the positive reappraisal had a protective effect for participants without a previous mental disorder. The possibility of changing the way of perceiving the pandemic, and seeking to find the positive aspects contribute to emotional regulation, decreasing the chances of experiencing stress and depression. Coping focused on emotion can have adaptive functions considering mechanisms mediated by optimism and analysis of the possibilities of controlling the problem (Gloria & Steinhardt, 2016).

Considering the protective effect of positive reappraisal demonstrates how cognitive strategies are efficient for stressful situations with a low level of direct control, confirming the principles of the transactional model of stress. The effect of strategies depends on the perception of problems and the assessment of resources available for coping. There are shreds of evidence that identify strategies focused on emotion as maladaptive, as they are ways to avoid or reduce the effects of stressors, and this may represent postponing the solution (Groth et al., 2019). On the other hand, there is evidence of adaptive processes demonstrated by the protective effect of positive reappraisal (Pirutinsky, Cherniak, & Rosmarin, 2020). Positive reappraisal works as a positive effect to deal with adverse life events. Cognitive adaptation is a more significant predictor of well-being when the dimensions involve positive thoughts and an optimistic perspective (Garnefski, van Rood, De Roos, & Kraaij, 2017).

Problem-solving was identified as a protective factor for depression (moderate and severe) and anxiety (severe). As it is a problem-focused strategy, more active behaviors are characterized by a plan to resolve stressful events using a collection of information about the stressful situation for decision making (Sawhney, Klinefelter, & Britt, 2018). Active coping strategies are less likely to be used under repeated stress conditions for long periods, and when the situation appears uncontrollable and unpredictable (Cantave et al., 2019).

Up to this moment, few studies have investigated the coping strategies used in the COVID-19 pandemic. Research developed with American Orthodox Jewish in the context of COVID-19 revealed that coping strategy focused on emotion, such as religiosity and belief in God, was correlated with less stress and a positive impact on mental health (Pirutinsky et al., 2020). Religious coping was also a strategy often used by the Malaysian population to deal with the COVID pandemic crisis (Perveen et al., 2020). Religious beliefs can be considered positive reappraisal strategies since religiosity allows an interpretation of the problem as something less harmful, and that can bring learning or more proximity to spirituality (Pirutinsky et al., 2020). A study carried out at U. K. found a mediating effect of flexibility on coping strategies. The ability to change perceptions about the problem and have flexibility explained 5–18% of the well-being variance and was inversely associated with avoidance strategies (Dawson & Golijani-Moghaddam, 2020). Humor was an effective strategy to help deal with pandemics’ adverse effects and help individuals have regular activities in the Malaysian population (Perveen et al., 2020). Regarding maladaptive coping strategies, another study developed in the UK identified that maladaptive coping responses partially mediated the predictive relationship between intolerance of uncertainty and psychological distress (Rettie & Daniels, 2020). Accepting responsibility is another coping focus on emotion found as a risk of moderate anxiety for people without previous mental disorder. This strategy contributes to self-blame and punitive behaviors (Stiegelis et al., 2003). The people believe that problems were caused by themselves. In patients with cancer, this strategy reduces actions promising the treatment (Miller, Manne, Taylor, Keates, & Dougherty, 1996).

People with previous mental disorders are recognized as more vulnerable to the adverse effects of the pandemic on mental health. The results showed that coping strategies in this group did not have a protective effect, which can be explained by the difficulty in developing strategies that are cognitive or even more active in dealing with the pandemic's consequences. In this group every coping strategy belongs to coping emotional focus, confrontation and escape. The effect of emotional coping as confrontation and escape were predictors of less mental health.

The pre-existence of mental disorders potentiates the stressful effects of a pandemic, mainly due to interruptions in mental health care routines and its association to increasing the potential for relapse or exacerbating symptoms (Chatterjee, Barikar, & Mukherjee, 2020; Druss, 2020; Yao et al., 2020). People with previous mental disorders had significantly higher scores on the COVID Stress Scales (Taylor et al., 2020). Individuals with anxiety-related disorders were more likely to isolate themselves and strive more actively to deal with the affliction of self-isolation, although there is no evidence of appreciable benefit from their coping methods (Asmundson et al., 2020). In addition to emotional vulnerability, in the study by Seminog and Goldacre (2013), it was identified that this group has a high risk of contagion of infectious diseases, such as the COVID-19 virus.

The evidence found contributes to public health actions during and after the outbreak. Strategies to mitigate the pandemic's harmful effects on mental health should consider the need for psychoeducational campaigns to stimulate the use of problem-focused strategies, such as problem-solving. However, strategies focused on emotion positive reappraisal are associated with cognitive mechanisms and are very appropriate for situations where people do not directly control the problem.

The research results must be interpreted considering some limitations: we analyzed a non-random sample, since the questionnaire was answered in a self-referenced and virtual way, once that face-to-face collection was not possible at this time of the pandemic. This data collection strategy could bias the sample that would be willing to fill the questionnaire, and thus it is not representative of the Brazilian population as a whole, especially in the context of different health-protective policies adopted by each city. Nevertheless, even though there is this variability of social distancing policies and pandemic peaks in different Brazilian states, no significant differences were found in the average days of social distancing practiced by the present study participants. Another limitation is that the study investigated the participants’ perception of their mental health and coping strategies. Since we only had self-perception mental health measures, one should interpret the results carefully because they are not specifically related to professionally diagnosed mental health disorders. This is a cross-sectional study; hence, we did not follow up on these participants’ previous mental health data that could influence their feeling through the pandemia.

## Conclusions

Practice of social distancing was a risk factor for worse mental health. This study's findings indicate that the coping strategies adopted may reduce the chances of depression, anxiety, and stress. These results have important implications for the planning of public policies to prevent and treat mental health problems that occurred during and after the pandemic. Participants with a previous mental disorder may need increased support to use more effective strategies to reduce stress, anxiety, and depression. Coping strategies presented a significant explanatory power to depression, anxiety, and stress and can be adopted by the population to reduce the pandemic's negative impact on mental health.

## Supplementary Material

Supplemental Material

## Data Availability

The data that support the findings of this study are available on request from the corresponding author. The data are not publicly available due to their containing information that could compromise research participants’ privacy or ethical restrictions.

## References

[CIT0001] Asmundson, G., Paluszek, M. M., Landry, C. A., Rachor, G. S., McKay, D., & Taylor, S. (2020). Do pre-existing anxiety-related and mood disorders differentially impact COVID-19 stress responses and coping? *Journal of Anxiety Disorders*, *74*, 102271. doi:10.1016/j.janxdis.2020.102271 32673930 PMC7342169

[CIT0002] Booker, J. A., Fivush, R., Graci, M. E., Heitz, H., Hudak, L. A., Jovanovic, T., … Stevens, J. S. (2020). Longitudinal changes in trauma narratives over the first year and associations with coping and mental health. *Journal of Affective Disorders*, *272*, 116–124. doi:10.1016/j.jad.2020.04.009 32379602 PMC7310038

[CIT0003] Browne-Yung, K., Walker, R. B., & Luszcz, M. A. (2017). An examination of resilience and coping in the oldest old using life narrative method. *The Gerontologist*, *57*(2), 282–291. doi:10.1093/geront/gnv137 26511273

[CIT0004] Campagne, D. M. (2019). Stress and perceived social isolation (loneliness). *Archives of Gerontology and Geriatrics*, *82*, 192–199. doi:10.1016/j.archger.2019.02.007 30825769

[CIT0005] Cantave, C. Y., Langevin, S., Marin, M. F., Brendgen, M., Lupien, S., & Ouellet-Morin, I. (2019). Impact of maltreatment on depressive symptoms in young male adults: The mediating and moderating role of cortisol stress response and coping strategies. *Psychoneuroendocrinology*, *103*, 41–48. doi:10.1016/j.psyneuen.2018.12.235 30640036

[CIT0006] Chatterjee, S. S., Barikar, C. M., & Mukherjee, A. (2020). Impact of COVID-19 pandemic on pre-existing mental health problems. *Asian Journal of Psychiatry*, *51*, 102071. doi:10.1016/j.ajp.2020.102071 32334407 PMC7165115

[CIT0007] Chew, Q. H., Wei, K. C., Vasoo, S., Chua, H. C., & Sim, K. (2020). Narrative synthesis of psychological and coping responses towards emerging infectious disease outbreaks in the general population: Practical considerations for the COVID-19 pandemic. *Singapore Psychiatry Research*, *61*, 350–356. doi:10.11622/smedj.2020046 PMC7926608 32241071

[CIT0008] Dang, A. K., Le, X., Le, H. T., Tran, B. X., Do, T., Phan, H., … Ho, R. (2020). Evidence of COVID-19 impacts on occupations during the first vietnamese national lockdown. *Annals of Global Health*, *86*(1), 112. doi:10.5334/aogh.2976 32944509 PMC7473180

[CIT0009] Dawson, D. L., & Golijani-Moghaddam, N. (2020). COVID-19: Psychological flexibility, coping, mental health, and wellbeing in the UK during the pandemic. *Journal of Contextual Behavioral Science*, *17*, 126–134. doi:10.1016/j.jcbs.2020.07.010 32834970 PMC7392106

[CIT0010] Dong, L., & Bouey, J. (2020). Public mental health crisis during COVID-19 pandemic, China. *Emerging Infectious Diseases*, *26*(7), 1616–1618. doi:10.3201/eid2607.200407 32202993 PMC7323564

[CIT0011] Druss, B. G. (2020). Addressing the COVID-19 pandemic in populations With serious mental illness. *JAMA Psychiatry*, *3*, e1–e2, 10.1001. doi:10.1001/jamapsychiatry.2020.0894 32242888

[CIT0012] El-Zoghby, S. M., Soltan, E. M., & Salama, H. M. (2020). Impact of the COVID-19 pandemic on mental health and social support among adult Egyptians. *Journal of Community Health*, *45*, 689–695. doi:10.1007/s10900-020-00853-5 32468155 PMC7255077

[CIT0013] Endler, Norman S., & Parker, James D. A. (1990). State and trait anxiety, depression and coping styles. *Australian Journal of Psychology*, *42*(*2*), 207–220. 10.1080/00049539008260119

[CIT0014] Folkman, S, & Lazarus, R.S. (1980). An analysis of coping in a middle-aged community sample. *Journal of Health and Social behavior*, *21*, 219–239. 7410799

[CIT0015] Folkman, S., & Lazarus, R. S. (1985). If it changes it must be a process: Study of emotion and coping during three stages of a college examination. *Journal of Personality and Social Psychology*, *48*(1), 150–170. doi:10.1037/0022-3514.48.1.150 2980281

[CIT0016] Folkman, S., & Lazarus, Richard S. (1985). If it changes it must be a process: Study of emotion and coping during three stages of a college examination. *Journal of Personality and Social Psychology*, *48*(1), 150–170. 10.1037/0022-3514.48.1.150 2980281

[CIT0017] Folkman, S., Lazarus, R. L., Dunkel-Schetter, C., DeLongis, A., & Gruen, R. (1986). Dynamics of a stressful encounter: Cognitive appraisal, coping, and encounter outcomes. Journal of Personality and Social Psychology, *50*, 992–1003. 10.1037//0022-3514.50.5.992 3712234

[CIT0018] Garnefski, N., van Rood, Y., De Roos, C., & Kraaij, V. (2017). Relationships between traumatic life events, cognitive emotion regulation strategies, and somatic complaints. *Journal of Clinical Psychology in Medical Settings*, *24*(2), 144–151. doi:10.1007/s10880-017-9494-y 28508141 PMC5487746

[CIT0019] Gerhold, L. (2020). COVID-19: Risk perception and Coping strategies. *PsyArXiv*. 10.31234/osf.io/xmpk4

[CIT0020] Gloria, C. T., & Steinhardt, M. A. (2016). Relationships among positive emotions, coping, resilience and mental health. *Stress and Health*, *32*(2), 145–156. doi:10.1002/smi.2589 24962138

[CIT0021] Grey, I., Arora, T., Thomas, J., Saneh, A., Tohme, P., & Abi-Habib, R. (2020). The role of perceived social support on depression and sleep during the COVID-19 pandemic. *Psychiatry Research*, *293*, 113452. doi:10.1016/j.psychres.2020.113452 32977047 PMC7500407

[CIT0022] Groth, N., Schnyder, N., Kaess, M., Markovic, A., Rietschel, L., Moser, S., … Schmidt, S. J. (2019). Coping as a mediator between locus of control, competence beliefs, and mental health: A systematic review and structural equation modelling meta-analysis. *Behaviour Research and Therapy*, *121*, 103442. doi:10.1016/j.brat.2019.103442 31430689

[CIT0023] Haber, M. G., Cohen, J. L., Lucas, T., & Baltes, B. B. (2007). The relationship between self-reported received and perceived social support: A meta-analytic review. *American Journal of Community Psychology*, *39*(1), 133–144. doi:10.1007/s10464-007-9100-9 17308966

[CIT0024] Hao, F., Tan, W., Jiang, L., Zhang, L., Zhao, X., Zou, Y., … Tam, W. (2020). Do psychiatric patients experience more psychiatric symptoms during COVID-19 pandemic and lockdown? A case-control study with service and research implications for immunopsychiatry. *Brain, Behavior, and Immunity*, *87*, 100–106. doi:10.1016/j.bbi.2020.04.069 PMC7184991 32353518

[CIT0025] Heffer, T., & Willoughby, T. (2017). A count of coping strategies: A longitudinal study investigating an alternative method to understanding coping and adjustment. *PloS One*, *12*, e0186057. doi:10.1371/journal.pone.0186057 28982138 PMC5642021

[CIT0026] Holmes, E. A., O'Connor, R. C., Perry, V. H., Tracey, I., Wessely, S., Arseneault, L., … Bullmore, E. (2020). Multidisciplinary research priorities for the COVID-19 pandemic: A call for action for mental health science. *The Lancet. Psychiatry*, *7*, 547–560. doi:10.1016/S2215-0366(20)30168-1 32304649 PMC7159850

[CIT0027] Huang, Y., & Zhao, N. (2021). Mental health burden for the public affected by the COVID-19 outbreak in China: Who will be the high-risk group? *Psychology, Health & Medicine*, *26*(1), 23–34. doi:10.1080/13548506.2020.1754438 32286091

[CIT0028] Lazarus, R. S., & Folkman, S. (1984). *Stress, Appraisal, and Coping*. New York, NY: Springer.

[CIT0029] Le, H. T., Nguyen, D. N., Beydoun, A. S., Le, X. T. T., Nguyen, T. T., Pham, Q. T., … Ho, R. C. M. (2020). Demand for health information on COVID-19 among Vietnamese. *International Journal of Environmental Research and Public Health*, *17*(12), E4377. doi:10.3390/ijerph17124377. PMID: 32570819. 32570819 PMC7344690

[CIT0030] Lewis, S. (2016). Only the lonely. *Nature Reviews Neuroscience*, *17*(4), 198–199. doi:10.1038/nrn.2016.26 26935169

[CIT0031] Lovibond, P. F., & Lovibond, S. H. (1995). The structure of negative emotional states: Comparison of the depression anxiety Stress Scales (DASS) with the beck depression and anxiety inventories. *Behaviour Research and Therapy*, *33*(3), 335–343. doi:10.1016/0005-7967(94)00075-u 7726811

[CIT0032] Miller, D. L., Manne, S. L., Taylor, K., Keates, J., & Dougherty, J. (1996). Psychological distress and well-being in advanced cancer: The effects of optimism and coping. *Journal of Clinical Psychology in Medical Settings*, *3*(2), 115–130. doi:10.1007/BF01996132 24226639

[CIT0033] Moreira, P. S., Ferreira, S., Couto, B., Machado-Sousa, M., Fernandez, M., Raposo-Lima, C., … Morgado, P. (2020). Protective elements of mental health status during the COVID-19 outbreak in the Portuguese population. *MedRxiv*, 10.1101/2020.04.28.20080671 PMC7920474 33669453

[CIT0034] Moreno, C., Wykes, T., Galderisi, S., Nordentoft, M., Crossley, N., Jones, N., … Arango, C. (2020). How mental health care should change as a consequence of the COVID-19 pandemic. *The Lancet Psychiatry*, *7*(9), 813–824. doi:10.1016/S2215-0366(20)30307-2 32682460 PMC7365642

[CIT0035] Penley, J. A., Tomaka, J., & Wiebe, J. S. (2002). The association of coping to physical and psychological health outcomes: A meta-analytic review. *Journal of Behavioral Medicine*, *25*(6), 551–603. doi:10.1023/a:1020641400589 12462958

[CIT0036] Perveen, A., Hamzah, H., Ramlee, F., Othman, A., & Minhad, M. (2020). Mental health and coping response among Malaysian adults during COVID-19 pandemic movement control order. *Journal of Critical Reviews*, *7*(18), 653–660. doi:10.31838/jcr.07.18.90

[CIT0037] Pfefferbaum, B., & North, C. S. (2020). Mental health and the covid-19 pandemic. *New England Journal of Medicine*, *383*, 510–512. doi:10.1056/NEJMp2008017 32283003

[CIT0038] Pirutinsky, S., Cherniak, A. D., & Rosmarin, D. H. (2020). COVID-19, mental health, and religious coping among American Orthodox jews. *Journal of Religion and Health*, 1–14. doi:10.1007/s10943-020-01070-z 32705481 PMC7377309

[CIT0039] Rajkumar, R. P. (2020). COVID-19 and mental health: A review of the existing literature. *Asian Journal of Psychiatry*, *52*, 102066. doi:10.1016/j.ajp.2020.102066 32302935 PMC7151415

[CIT0040] Rettie, H., & Daniels, J. (2020). Coping and tolerance of uncertainty: Predictors and mediators of mental health during the COVID-19 pandemic. *American Psychologist*, doi:10.1037/amp0000710 32744841

[CIT0041] Roy, D. (2020). Study of knowledge, attitude, anxiety & perceived mental healthcare need in Indian population during COVID-19 pandemic. *Asian Journal of Psychiatry*, doi:10.1016/j.ajp.2020.1020835 PMC7139237 32283510

[CIT0042] Savóia, M. G., Santana, P. R., & Mejias, N. P. (1996). Adaptação do inventário de estratégias de coping de Folkman e Lazarus para o português. *Psicologia usp*, *7*(1-2), 183–201. doi:10.1590/S1678-51771996000100009

[CIT0043] Sawhney, G., Klinefelter, Z., & Britt, T. W. (2018). Integrating coping and recovery: Review and recommendations for future research. *Journal of Applied Biobehavioral Research*, *23*, e12156. doi:10.1111/jabr.12156

[CIT0044] Seminog, O. O., & Goldacre, M. J. (2013). Risk of pneumonia and pneumococcal disease in people with severe mental illness: English record linkage studies. *Thorax*, *68*, 171–176. doi:10.1136/thoraxjnl-2012-202480 23242947

[CIT0045] Stiegelis, H. E., Hagedoorn, M., Sanderman, R., Van der Zee, K. I., Buunk, B. P., & Van den Bergh, A. C. (2003). Cognitive adaptation: A comparison of cancer patients and healthy references. *British Journal of Health Psychology*, *8*(3), 303–318. doi:10.1348/135910703322370879 14606975

[CIT0046] Tan, W., Hao, F., McIntyre, R. S., Jiang, L., Jiang, X., Zhang, L., … Zhang, Z. (2020). Is returning to work during the COVID-19 pandemic stressful? A study on immediate mental health status and psychoneuroimmunity prevention measures of Chinese workforce [published online ahead of print, 2020 Apr 23]. *Brain, Behavior, and Immunity*, S0889-1591(20)30603-6. doi:10.1016/j.bbi.2020.04.055 PMC7179503 32335200

[CIT0047] Taylor, S., Landry, C. A., Paluszek, M. M., Fergus, T. A., McKay, D., & Asmundson, G. J. (2020). COVID stress syndrome: Concept, structure, and correlates. *Depression and Anxiety*, *37*(8), 706–714. doi:10.1002/da.23071 32627255 PMC7362150

[CIT0048] Taylor, S. E., & Stanton, A. L. (2007). Coping resources, coping processes, and mental health. *Annual Review of Clinical Psychology*, *3*, 377–401. doi:10.1146/annurev.clinpsy.3.022806.091520 17716061

[CIT0049] Tee, M. L., Tee, C. A., Anlacan, J. P., Aligam, K. J. G., Reyes, P. W. C., Kuruchittham, V., & Ho, R. C. (2020 August 24). Psychological impact of COVID-19 pandemic in the Philippines. *Journal of Affective Disorders*, *277*, 379–391. doi:10.1016/j.jad.2020.08.043. Epub ahead of print. PMID: 32861839. 32861839 PMC7444468

[CIT0050] Torales, J., O'Higgins, M., Castaldelli-Maia, J. M., & Ventriglio, A. (2020). The outbreak of COVID-19 coronavirus and its impact on global mental health. *International Journal of Social Psychiatry*, *66*, 317–320. doi:10.1177/0020764020915212 32233719

[CIT0051] Tran, B. X., Nguyen, H. T., Le, H. T., Latkin CA, Pham HQ, Vu LG, Le XT, Nguyen TT, Pham QT, Ta NT. (2020). Impact of COVID-19 on economic well-being and quality of life of the Vietnamese during the national social distancing. *Frontier is Psychology*, *11*, 565153. doi:10.3389/fpsyg.2020.565153. PMID: 33041928; PMCID: PMC7518066. PMC7518066 33041928

[CIT0052] Twenge, J., & Joiner, T. (2020). Mental distress among US adults during the COVID-19 pandemic. *PsyArXiv*. 10.31234/osf.io/wc8ud PMC7675251 33037608

[CIT0053] Vignola, R. C. B., & Tucci, A. M. (2014). Adaptation and validation of the depression, anxiety and stress scale (DASS) to Brazilian Portuguese. *Journal of Affective Disorders*, *155*, 104–109. doi:10.1016/j.jad.2013.10.031 24238871

[CIT0054] Wang, C., Pan, R., Wan, X., Tan, Y., Xu, L., Ho, C. S., & Ho, R. C. (2020a). Immediate psychological responses and associated factors during the initial stage of the 2019 coronavirus disease (COVID-19) epidemic among the general population in China. *International Journal of Environmental Research and Public Health*, *17*(5), 1729. Published 2020 Mar 6. doi:10.3390/ijerph17051729. PMC7084952 32155789

[CIT0055] Wang, C., Pan, R., Wan, X., Tan, Y., Xu, L., McIntyre, R. S., … Ho, C. (2020b). A longitudinal study on the mental health of general population during the COVID-19 epidemic in China [published online ahead of print, 2020 Apr 13]. *Brain, Behavior, and Immunity*, *2020*, S0889-1591(20)30511-0. doi:10.1016/j.bbi.2020.04.028 PMC7153528 32298802

[CIT0056] Wang, H., Xia, Q., Xiong, Z., Li, Z., Xiang, W., Yuan, Y., … Li, Z. (2020). The psychological distress and coping styles in the early stages of the 2019 coronavirus disease (COVID-19) epidemic in the general mainland Chinese population: A web-based survey. *Plos one*, *15*(5), e0233410. doi:10.1371/journal.pone.0233410 32407409 PMC7224553

[CIT0057] Wu, M., Xu, W., Yao, Y., Zhang, L., Guo, L., Fan, J., & Chen, J. (2020). Mental health status of students’ parents during COVID-19 pandemic and its influence factors. *General Psychiatry*, *33*(4). doi:10.1136/gpsych-2020-100250 PMC7387315 34192232

[CIT0058] Xiao, H., Zhang, Y., Kong, D., Li, S., & Yang, N. (2020). The effects of social support on sleep quality of medical staff treating patients with coronavirus disease 2019 (COVID-19) in January and February 2020 in China. *Medical Science Monitor*, *26*, e923549. doi:10.12659/MSM.923549 32132521 PMC7075079

[CIT0059] Xiong, J., Lipsitz, O., Nasri, F., Lui, L., Gill, H., Phan, L., … McIntyre, R. S. (2020). Impact of COVID-19 pandemic on mental health in the general population: A systematic review. *Journal of Affective Disorders*, *277*, 55–64. doi:10.1016/j.jad.2020.08.001 32799105 PMC7413844

[CIT0060] Yao, H., Chen, J. H., & Xu, Y. F. (2020). Patients with mental health disorders in the COVID-19 epidemic. *The Lancet Psychiatry*, *7*(4), e21. doi:10.1016/S2215-0366(20)30090-0 32199510 PMC7269717

[CIT0061] Zhang, Y., & Ma, Z. F. (2020). Impact of the COVID-19 pandemic on mental health and quality of life among local residents in liaoning province, China: A cross-sectional study. *International Journal of Environmental Research and Public Health*, *17*(7), 2381. doi:10.3390/ijerph17072381 PMC7177660 32244498

